# Quality of glycemic control in type 2 diabetes mellitus (T2DM) and its association with markers of coagulation and inhibitors of fibrinolysis: A case–control study in the Upper West Region, Ghana

**DOI:** 10.1002/hsr2.1297

**Published:** 2023-06-07

**Authors:** Peter K. Selleh, Enoch O. Anto, Wina I. O. Boadu, Benedict Sackey, Lilian A. Boateng, Charles Nkansah, Frederick Nsafoah, Abdul R. Saasi, Selina Mintaah, Yaw A. Wiafe, Charles Derigubah, Emmanuel E. Korsah, Joseph Frimpong, Ezekiel Ansah, Valentine C. K. T. Tamakloe, Patrick Adu, Joseph Boachie, Otchere Addai‐Mensah

**Affiliations:** ^1^ Department of Medical Diagnostics, Faculty of Allied Health Sciences, College of Health Sciences Kwame Nkrumah University of Science and Technology Kumasi Ghana; ^2^ School of Medical and Health Sciences Edith Cowan University Joondalup Western Australia Australia; ^3^ Centre for Precision Health, ECU Strategic Research Centre Edith Cowan University Joondalup Western Australia Australia; ^4^ Department of Medical Laboratory Science, School of Allied Health Sciences University of Cape Coast Cape Coast Ghana

**Keywords:** coagulation, glycemic control, plasminogen activator inhibitor, thrombin activatable fibrinolysis inhibitor, type 2 diabetes mellitus

## Abstract

**Background and Aims:**

Type 2 diabetes mellitus (T2DM) individuals are at a higher risk of developing diabetes complications, with approximately 80% complication‐related mortality. The increased morbidity and mortality among T2DM patients are partly due to dysregulated hemostasis. This study determined the quality of glycemic control in T2DM and its association with markers of coagulation and inhibitors of fibrinolysis.

**Methods:**

This case–control study recruited 90 participants involving: 30 T2DM patients with good glycemic control, 30 with poor glycemic control, and 30 nondiabetic subjects as controls at a Municipal Hospital in Ghana. Fasting blood glucose, glycated hemoglobin, activated partial thromboplastin time (APTT), prothrombin time (PT), calculated international normalized ratio (INR), and full blood count (FBC) were determined for each respondent. Plasma levels of plasminogen activator inhibitor‐1 (PAI‐1) and thrombin activatable fibrinolysis inhibitor (TAFI) were determined using the solid‐phase sandwich enzyme‐linked immunosorbent assay method. Data were analyzed using R language software.

**Results:**

Plasma PAI‐1 antigen levels were significantly higher in the participants with poor glycemic control as compared to participants with good glycemic control (*p* < 0.0001). There was no significant difference in plasma TAFI levels between the participants with poor glycemic control as compared to participants with good glycemic control (*p* = 0.900). T2DM patients had significantly shorter APTT, PT, and INR than controls (*p* < 0.05). At a cut‐off of ≥161.70 pg/μL, PAI was independently associated with increasing odds (adjusted odds ratio = 13.71, 95% confidence interval: 3.67–51.26, *p* < 0.0001) of poor glycemic control and showed the best diagnostic accuracy for poor glycemic control (area under the curve = 0.85, *p* < 0.0001).

**Conclusion:**

PAI‐1 levels were significantly increased in T2DM with poor glycemic control and emerged as the best predictor for poor glycemic control. Good glycemic management to control the plasma levels of PAI‐1 is required to prevent hypercoagulability and thrombotic disorders.

## 1 INTRODUCTION

Diabetes mellitus (DM) is a metabolic disorder characterized by persistent hyperglycemia triggered by the pancreas’ inability to make enough insulin (type 1 DM) or the body's inability to efficiently utilize the insulin it produces (type 2 DM) or both. It may manifest as polyuria, frequent thirst, polyphagia, blurred vision, and weight loss. Globally, T2DM constitutes about 90%–95% of DM cases diagnosed.^1^

Nearly 80% of patients with T2DM die due to complications associated with the disease. The increased morbidity and mortality among T2DM patients are partly due to dysregulated hemostasis.^2^, ^3^, ^4^ T2DM patients have an aggregate thrombotic risk including hyperreactivity of platelets, upregulation of prothrombotic markers, and a decrease in fibrinolysis. These alterations are primarily mediated by insulin resistance, endothelial dysfunction, dysglycemia, and an elevated inflammatory state, all of which have a direct impact on platelet function, coagulation factors, and clot formation.^5^, ^6^, ^7^ The glycosylation of essential proteins and the upsurge in plasma levels of some clotting factors contribute to an increased clotting tendency in T2DM.^8^, ^9^ A good glycemic control (glycated hemoglobin [HbA1c] <7.0%) in T2DM prevents or slows down the progression of complications.^10^, ^11^ Glycemic control is thus considered as the main therapeutic goal for the prevention of complications in T2DM.

The antifibrinolytic proteins such as thrombin activatable fibrinolysis inhibitor (TAFI), plasminogen activator inhibitor‐1 (PAI‐1), and α‐2‐antiplasmin play crucial roles in the hemostatic pathway by preventing the premature breakdown of a fibrin clot at the site of endothelial injury. These unique proteins function to inhibit the activators of plasminogen to plasmin and also directly inhibit plasmin.^12^ T2DM induces inflammation, with the associated release of cytokines upregulating antifibrinolytics, especially PAI‐1.^13^, ^14^ Increased plasma levels of these inhibitors of fibrinolysis can adversely affect fibrin clot breakdown leading to a hypofibrinolytic state that may partly contribute to the incidence of thrombotic disorders as seen in T2DM.^15^, ^16^ It is thus imperative to evaluate the diagnostic accuracy of PAI‐1 and TAFI and its relation to glycemic control among T2DM patients as failure to timely detect the changes in fibrinolysis predisposes to thromboembolism, which increases mortality in these patients.

To the best of our knowledge, no study has assessed these antifibrinolytics among T2DM patients in Ghana. The study by Ephraim et al.^8^ was confined to only coagulation tendency but could not determine the impact of poor glycemic control on coagulation and the plasma levels of PAI‐1 and TAFI. Also, Nkansah et al.^17^ limited their study to plasma antigen and activity levels of PAI‐1 in T2DM.^17^ This study therefore aimed at comparing the markers of coagulation and inhibitors of fibrinolysis in T2DM patients relative to the quality of glycemic control in Northern Ghana.

## 2 MATERIALS AND METHODS

### 2.1 Study design and site

This hospital‐based case–control study was conducted in the Diabetes Outpatient Clinic of the Wa Municipal Hospital in the Upper West Region between January 2021 and August 2021. Wa is one of the major cities in Northern Ghana and has an estimated population of about 107,214 representing 15.3% of the regional population. It is located in the North‐Western part of the country, between latitudes 1°40′–2°45′ N and longitude 9°32′–10°20′ W.^18^

### 2.2 Selection of study subjects

The study population included T2DM subjects attending the Diabetic Clinic of Wa Municipal Hospital as the case group and apparently healthy nondiabetic blood donors as the control group. T2DM patients who had had the condition for at least 1 year were recruited. This study excluded T1DM individuals and expectant women. T2DM clients undergoing dialysis, on blood thinners, with bleeding disorders, cerebrovascular and peripheral vascular diseases, and liver or kidney function impairment were excluded from the study. T2DM patients and blood donors with ages ranging from 30 to 80 years were recruited into the study. A well‐structured questionnaire was used to obtain sociodemographic data from participants, while supplementary clinical history and other relevant data were obtained from participants' medical records.

### 2.3 Ethics and participant's consent

Ethical approval was obtained from the Committee on Human Research, Publication and Ethics (CHRPE) of the Kwame Nkrumah University of Science and Technology (Ref. no.: CHRPE/AP/092/21). All participants gave written informed consent.

### 2.4 Sample size justification

The sample size was determined by using Kelsey's formula as follows:

$$N_{\mathrm{cases}-\mathrm{Kelsey}}=\left[\frac{r+1}{r}\right]\frac{p(1-P){\left(Z_{\frac{\alpha }{2}}+Z_{\beta }\right)}^{2}}{{\left(p_{1}-p_{2}\right)}^{2}}\;\;\text{and}\;\;P=\left[\frac{p_{1}+\left(rXp_{2}\right)}{r+1}\right],$$

where *N*_cases–Kelsey_ is the required sample size for the T2DM group; *r* is the ratio of nondiabetics to T2DM, which is 1:2 in this study; $Z_{\frac{\alpha }{2}}$ represents the critical value of the normal dispersion at *α*/2 (for this study at a confidence level of 95%; *α* is 0.05 and the critical value is 1.96). $Z_{\beta }$ represents the critical value of the normal distribution at *β* (this study used a power of 80%; *β* is 0.2 and the critical value is 0.84); *p*_1_ represents the percentage of the risk of thrombosis in the diabetic group, 40% according to Tsai et al.^19^; *p*_2_ is the proportion of the nondiabetic group at risk of thrombosis, 10.7% according to Piazza et al.^20^; *p*_1_ − *p*_2_ is the smallest difference in proportions that is clinically important.

From the formula above, the minimum number of T2DM patients required for this study was 58 as against 29 non‐DM individuals. However, this study recruited 90 subjects: 60 T2DM subjects comprising 30 T2DM persons with good glycemic control and 30 with poor glycemic control and 30 nondiabetic blood donors.

### 2.5 Anthropometric and blood pressure measurements

Weight was measured in the upright position to the nearest 0.1 kg using a calibrated balance beam scale. Height was measured in centimeters (cm) using a measuring tape and converted to the nearest 0.1 m. The body mass index (BMI) was computed by dividing the recorded weight in kilograms by the height in meters squared (kg/m^2^). After sitting for at least 5 min; a subject's blood pressure (BP) was measured from the right arm using an automatic BP machine—Omron MX3 (Omron Corporation). Two readings were recorded 5 min apart and the average of the two results was determined. A systolic BP of 140 mmHg or greater or a diastolic BP of 90 mmHg or greater, or a history of the previously known disease was considered hypertension.^21^

### 2.6 Blood sample collection and biochemical assay

Six milliliters of fasting venous blood were taken aseptically from the antecubital fossa region with participants seated. Fasting blood glucose was determined immediately using an Accu‐Check Aviva Plus glucometer (Roche Diabetes Care Inc.) while 3.6 mL of blood was dispensed into a tube containing 380 μL of 3.2% buffered trisodium citrate and the remaining blood was dispensed into K3‐EDTA tube for platelet count using the 5‐part differential Sysmex FBC analyzer (Sysmex Inc.), and glycated hemoglobin (HbA1c) was estimated using multipurpose Finecare hormonal analyzer (Wondfo Biotech Co. Ltd.). The plasma from the trisodium citrate sample was used for the estimation of activated partial thromboplastin time (APTT), prothrombin time (PT), and international normalized ratio (INR) using Sysmex semiautomated coagulation blood analyser (Model CA‐104) at the Kaara Diagnostics Laboratory. All other sample analyses were done at the St Joseph's Hospital Laboratory in Jirapa in the Upper West Region. The remaining K3‐EDTA sample was put in a centrifuge and spun at 3000 rpm for 10 min to obtain the plasma. The plasma samples were transferred into Eppendorf tubes, labelled, and refrigerated at −20°C (for 1 month) until we received the ELISA (enzyme‐linked immunosorbent assay) test kits. The measurement of PAI‐1 and TAFI antigen levels was done by the solid‐phase sandwich ELISA technique using the Thermo Electron Multiskan EX plate reader.

### 2.7 Definition of quality of glycemic control

Glycemic control was classified as poor or good. Poor control of diabetes was defined as HbA1c ≥ 7% according to the guidelines on glycemic targets for diabetes control, while good glycemic control was defined as HbA1c < 7%.^22^

### 2.8 Data analysis

Data were recorded and filtered in Microsoft Excel 2010 and analysis was done using R language software (R version 4.2.1). Categorical data were presented in frequencies with corresponding percentages within parentheses, while continuous data were presented as median (interquartile ranges). *χ* ^2^ and Fisher's exact tests were used appropriately to compare categorical variables and continuous data were compared using Mann–Whitney *U*‐test. Logistic regression analysis was used to establish the effect and predictive relationships among variables. The receiver‐operating characteristic (ROC) analysis was used to determine the diagnostic accuracies of the markers. *p* Values less than 0.05 were considered statistically significant.

## 3 RESULTS

### 3.1 Baseline characteristics of participants

Table 1 shows the baseline characteristics of the study groups. A total of 90 participants consisting of 60 T2DM patients (cases) and 30 healthy blood donors (controls) were included in the statistical analysis. The females constituted a greater percentage of both the control (53.3%) and the T2DM (78.3%) groups in this study. Most T2DM patients did not get formal education (56.7%) as compared to controls (10.0%), with a greater percentage of the T2DM patients being self‐employed (36.7%). Most T2DM patients did not take alcohol (65.0%). The participants with T2DM (cases) were older compared to the healthy controls [59.50 years (52.25–65.00) vs. 37.00 years (34.00–41.00), *p* < 0.0001]. Again, the T2DM individuals had significantly higher BMI (23.71 vs. 27.85, *p* = 0.0080), SBP (98.00 vs. 124.00, *p* < 0.0001), HbA1c (5.15 vs. 6.85, *p* < 0.0001), and FBG (5.00 vs. 6.60, *p* < 0.0001) compared to the controls. There was no significant difference in DBP between the cases and controls (*p* > 0.05).

**Table 1:** Baseline characteristics of participants.

Variable	Non‐T2DM (%) (*n* = 30)	T2DM (%) (*n* = 60)	*p* Value
Gender			**0.0267**
Male	14 (46.7)	13 (21.7)	
Female	16 (53.3)	47 (78.3)	
Educational level			**<0.0001**
No formal education	3 (10.0)	34 (56.7)	
Primary/basic school	8 (26.7)	6 (10.0)	
Secondary school	8 (26.7)	12 (20.0)	
Tertiary	11 (36.7)	8 (13.3)	
Occupation			**0.0040**
Unemployed	7 (23.3)	18 (30.0)	
Self‐employed	7 (23.3)	22 (36.7)	
Employed	16 (53.3)	11 (18.3)	
Retired	0 (0.0)	9 (15.0)	
Alcohol Intake			**<0.0001**
No	7 (23.3)	39 (65.0)	
Yes	23 (76.7)	21 (35.0)	
Age (years)	37.00 (34.00–41.00)	59.50 (52.25–65.00)	**<0.0001**
BMI (kg/m^2^)	23.71 (22.07–27.12)	27.85 (23.85–32.04)	**0.0080**
SBP (mmHg)	98.00 (90.00–102.00)	124.00 (111.00–140.00)	**<0.0001**
DBP (mmHg)	75.00 (70.00–80.00)	77.00 (65.25–86.50)	0.9350
FBG (mmol/L)	5.00 (4.50–5.53)	6.60 (5.63–8.63)	**<0.0001**
HBA1c (%)	5.15 (4.63–5.83)	6.85 (6.40–10.10)	**<0.0001**

*Note*: *p* Values for categorical variables were computed by *χ* ^2^ and Fisher's exact tests and for continuous variables by Mann–Whitney *U*‐test.

Abbreviations: BMI, body mass index; DBP, diastolic blood pressure; FBG, fasting blood glucose; HBA1c, glycated hemoglobin; SBP, systolic blood pressure.

### 3.2 Levels of PAI‐1, TAFI, and other basic coagulation parameters among study participants

**Figure 1:**
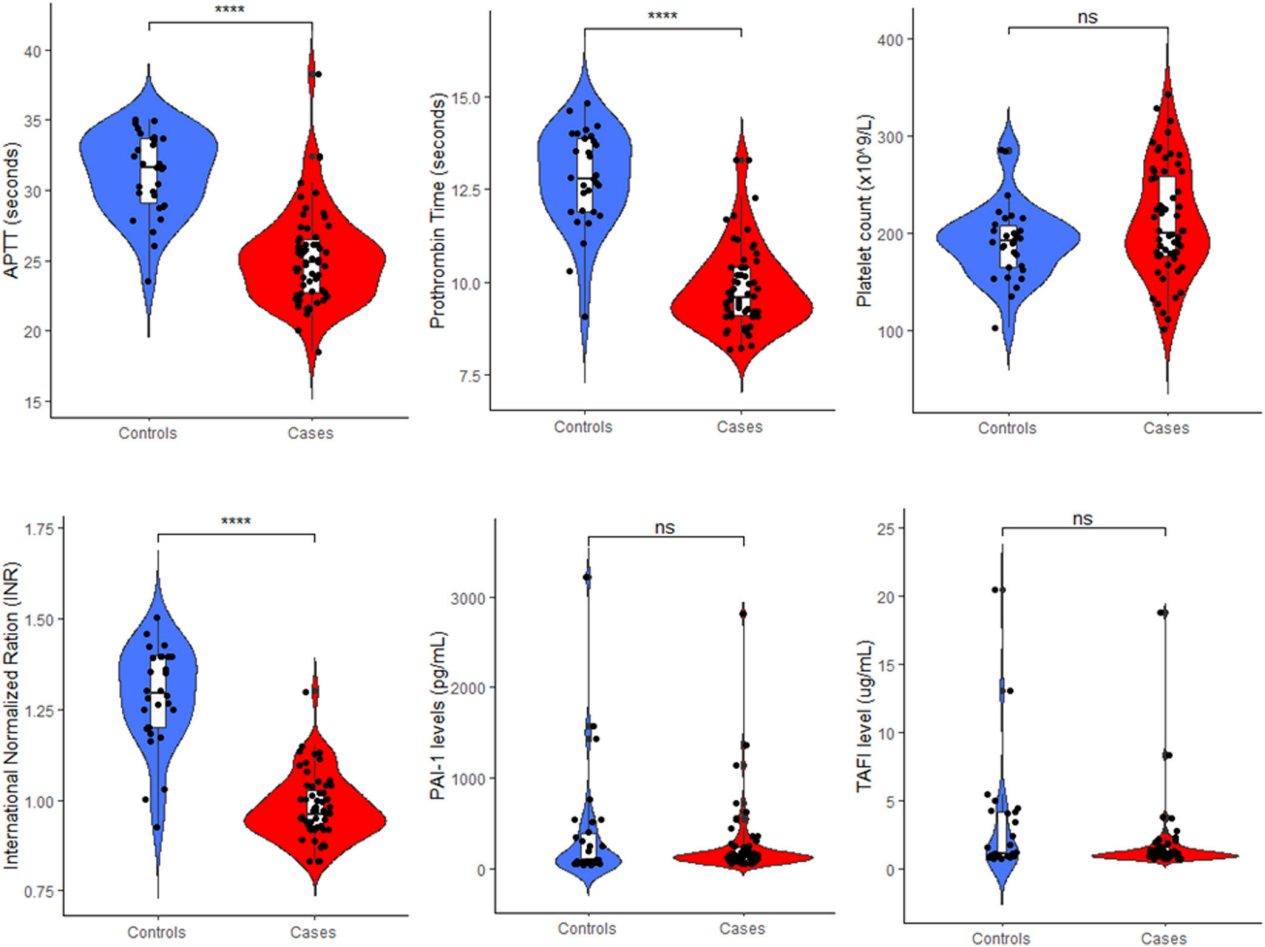
Levels of plasminogen activator inhibitor type 1 (PAI‐1), thrombin activatable fibrinolysis inhibitor (TAFI), and other basic coagulation parameters among type 2 diabetes mellitus (T2DM) patients and healthy controls. APTT, activated partial thromboplasmin time; PT, prothrombin time. *p* Values computed by Mann–Whitney *U*‐test; ns, *p* > 0.05; ****p < 0.0001.

The T2DM patients (cases) had significantly lower (shortened) APTT, PT, and INR compared to the non‐DM (controls) (*p* < 0.0001). However, platelet count and levels of PAI‐1 and TAFI were not significantly different between cases and controls (*p* > 0.05) (Figure 1).

### 3.3 Levels of PAI‐1, TAFI, and other basic coagulation parameters among T2DM patients with good and poor glycemic control

**Figure 2:**
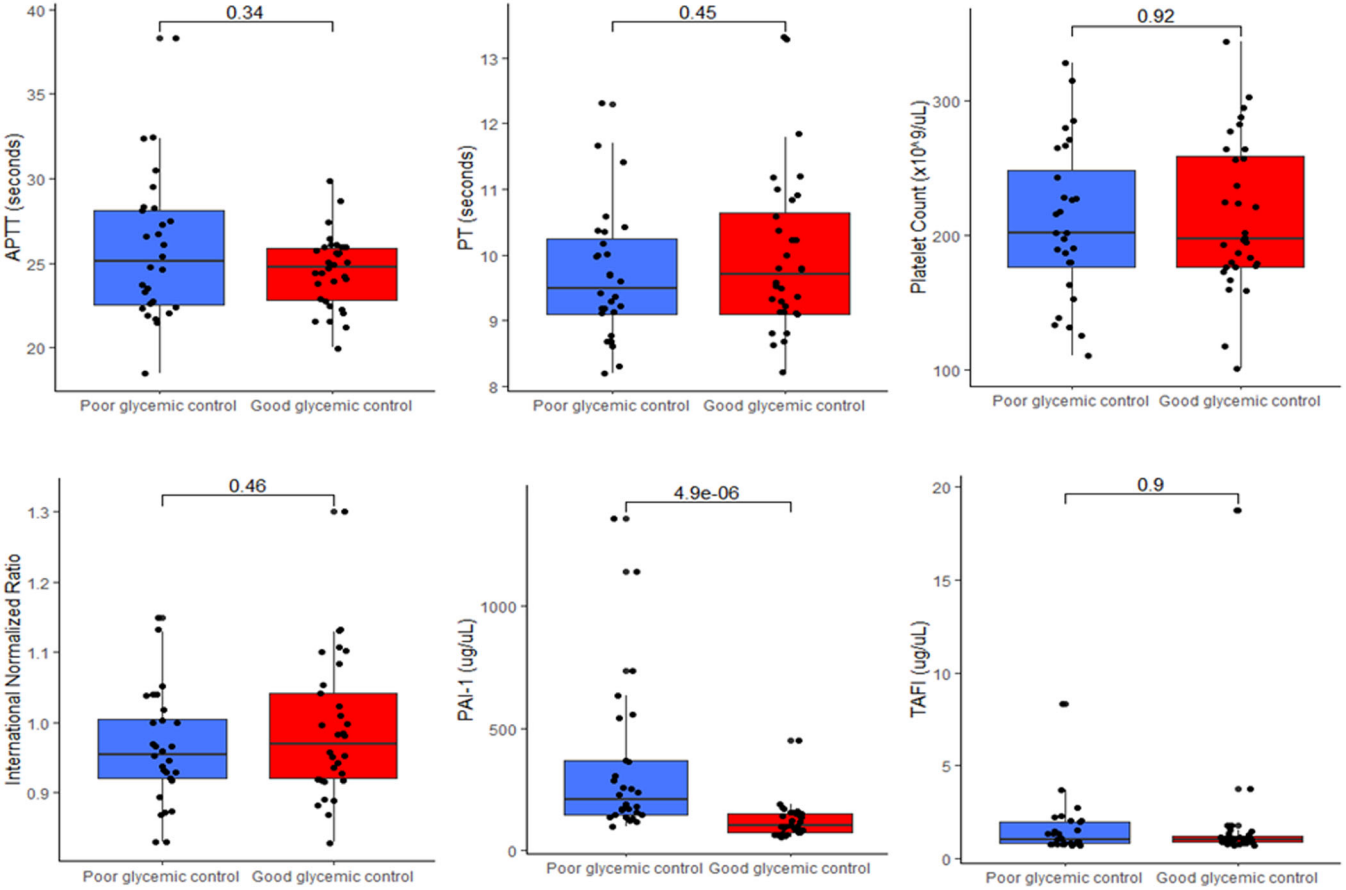
Levels of plasminogen activator inhibitor type 1 (PAI‐1), thrombin activatable fibrinolysis inhibitor (TAFI), and other basic coagulation parameters among type two diabetes patients stratified by glycemic control. APTT, activated partial thromboplasmin time; PT, prothrombin time. *p* Values were computed by Mann–Whitney *U*‐test.

Figure 2 displays the levels of PAI‐1, TAFI, and other basic coagulation parameters among T2DM patients with good and poor glycemic control. Except for PAI‐1 antigen level that was significantly elevated in poorly controlled T2DM patients (*p* < 0.0001), there were no statistically significant differences in the levels of TAFI, APTT, PT, INR, and platelet count between T2DM patients with poor glycemic control and those with good glycemic control (*p* > 0.05).

### 3.4 Diagnostic accuracies of PAI‐1 and TAFI in detecting poor glycemic control among T2DM patients

**Figure 3:**
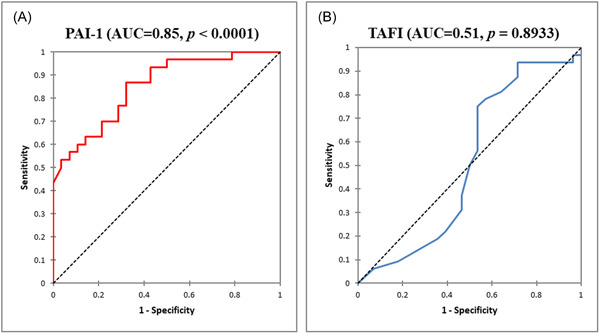
Receiver‐operating characteristics (ROCs) of plasminogen activator inhibitor type 1 (PAI‐1) (A) and thrombin fibrinolysis inhibitor (TAFI) (B) for predicting poor glycemic control among type 2 diabetes mellitus patients. AUC, area under the curve.

The diagnostic accuracies of PAI‐1 and TAFI were assessed in a ROC analysis. PAI‐1 was the best marker for predicting poor glycemic control among T2DM patients. At a cut‐off value of ≥161.70 pg/μL, PAI‐1 had a high (*p* < 0.0001) discriminating power of 85% (area under the curve [AUC] = 0.85) (Figure 3A), a sensitivity of 86.7%, a specificity of 67.8%, a negative predictive value of 82.6% and a positive predictive value of 74.3%. Type 2 diabetes patients who had PAI‐1 levels of ≥161.70 pg/μL had over 13‐fold increased odds of having poor glycemic control (adjusted odds ratio [aOR] = 13.70, 95% confidence interval [CI]: 3.67–51.26, *p* < 0.0001] (Table 2). On the other hand, TAFI could not significantly predict poor glycemic control among type 2 diabetics (*p* = 0.8933), with a very low discriminating power of 51% (AUC = 0.51) (Figure 3B). At a cut‐off value of ≥1.80 μg/μL, TAFI had a higher sensitivity (93.7%) but a very low specificity (28.6%) (Table 2).

**Table 2:** Accuracies of PAI‐1 and TAFI in detecting poor glycemic control among type 2 diabetes mellitus patients.

Marker	Cut‐off	Sensitivity (%)	Specificity (%)	PPV	NPV	aOR (95% CI)	*p* Value
PAI‐1	≥161.70 pg/μL	86.7 (69.5–95.2)	67.8 (55.2–82.1)	74.3	82.6	13.71 (3.67–51.26)	**<0.0001**
TAFI	≥1.80 μg/μL	93.7 (78.6–99.1)	28.6 (15.2–47.3)	60.0	80	2.80 (0.74–10.59)	0.1290

*Note*: Adjustments were made for age, gender, educational level, and hypertension. Association with significant *p*‐values in bold.

Abbreviations: aOR, adjusted odds ratio; CI, confidence interval; NPV, negative predictive value; PAI‐1, plasminogen activator inhibitor type 1; PPV, positive predictive value; TAFI, thrombin activatable fibrinolysis inhibitor.

## 4 DISCUSSION

T2DM patients are predisposed to microvascular‐ and macrovascular‐related complications such as cardiovascular disorders, diabetic retinopathy, neuropathy, and nephropathy.^23^ Some of these disorders have been attributed to an imbalance in the hemostatic and thrombosis‐protecting systems, resulting in a hypercoagulable state.^24^ This hypercoagulability shifts the thrombohemorheological balance in favor of thrombosis with a consequent shortening of APTT and PT. Reduced fibrinolysis in T2DM may be due to an increase in the antifibrinolytic antigens—TAFI and PAI‐1—in plasma. Whereas TAFI is a glycoprotein secreted by the hepatocytes, PAI‐1 is produced by various cells of the body such as the platelets, adipocytes, hepatocytes, endothelial cells, and so on, and both inhibit fibrinolysis by decreasing the tPA/plasminogen binding sites in fibrin and direct inhibition of the activators of plasminogen, respectively.^25^

In this study, PAI‐1 plasma levels were significantly increased in T2DM patients with poor glycemic control and PAI‐1 emerged as a good predictor of poor glycemic control with a high specificity and sensitivity of diagnostic accuracy. This finding suggests that poorly controlled T2DM individuals could be at a greater risk of thrombotic disorder particularly due to hypofibrinolysis as depicted by the increased PAI‐1 observed in the present study. The finding agrees with other studies conducted among T2DM patients.^17^, ^25^

Hyperglycemia‐induced oxidative stress is thought to raise proinflammatory protein levels with infiltrating macrophages secreting inflammatory cytokines resulting in local and systemic inflammation. PAI‐1 is said to act as an acute‐phase protein and therefore levels rise in plasma during inflammation in response to inflammatory cytokines (tumor growth factor‐β, interleukin‐1β, and tumor necrosis factor‐α).^7^ Additionally, hyperglycemia and hypertriglyceridemia are metabolic diseases induced by either relative or absolute insulin insufficiency. It is suggested that increased glucose concentrations stimulate PAI‐1 expression in endothelial and vascular smooth muscle cells in vitro. Also, it is suggested that triglycerides and their constituents (fatty acids) also stimulate PAI‐1 expression in HepG2 cells.^26^ As a result, increased PAI‐1 production in T2DM may be due to a combination of variables related to hyperglycemia and insulin resistance.

The plasma levels of PAI‐1 were, however, not significantly different between controls and T2DM patients in our study. On the contrary, Nkansah et al.^17^ in their study recorded a significantly higher plasma level of PAI‐1 antigen and activity in T2DM patients than controls, and this disparity could be due to genetic variations between our study groups as other genetic studies indicated that polymorphisms in PAI‐1 gene (genetic variants) influence the plasma levels of the antigen.^27^, ^28^ The probable explanation for our study finding could also imply that the medications used in the management of T2DM patients may have beneficial impacts in limiting the plasma levels of antifibrinolytics. In our present study, a majority of T2DM individuals were on metformin treatment—a biguanide known to reduce the plasma levels of PAI‐1. Metformin not only attenuates the secretion of insulin and enhanced insulin sensitivity but also attenuates the precursors of insulin that have been known to stimulate the expression of PAI‐1 in vivo.^29^

Another finding of the present study was that plasma levels of TAFI did not significantly differ between the T2DM cases and non‐T2DM controls. Also, the quality of glycemic control—good or bad—did not influence plasma levels of TAFI among T2DM patients. This finding is contrary to previous case–control studies^30^, ^31^ that established a significant difference in plasma levels of TAFI when T2DM cases were compared to non‐T2DM controls. The disparity in our study results could be attributed to genetic and geographic differences between study participants.

Our findings show a significantly shorter APTT and PT and a reduced INR among T2DM patients as compared to non‐T2DM controls. The implication is that T2DM patients are at higher risk of coagulation disorders, a finding in the present study, which is similar to the findings of Ephraim et al.^8^ The coagulation risk is due to the general impact of insulin resistance and hyperglycemia‐induced oxidative stress that culminates in endothelial dysfunction leading to increased coagulation tendency in T2DM.

Conversely, there was no statistically significant difference in APTT, PT, and INR when T2DM patients were compared based on the quality of glycemic control. Besides, platelet count was neither significantly different when T2DM cases were compared to non‐T2DM controls nor when good glycemic controls were compared to poor glycemic controls. This finding is similar to the results of previous studies conducted in Ghana among T2DM patients.^8^, ^17^ However, a case–control study in Nigeria recorded a significantly increased mean platelet count in T2DM patients than in nondiabetic controls.^32^ The dysglycemia, increased inflammatory state, and endothelial dysfunction associated with T2DM are believed to promote increases in plasma levels of platelets. This variation in our research findings compared to the previous study in Nigeria could be attributed to the differences in sample sizes.

Despite the interesting findings in the present study, there were some limitations, especially the inability to measure the panel of the fibrinolytic system and also assess the polymorphisms of the antifibrinolytics. Despite these limitations, our study indicates that the level of PAI‐1 antigen is a good predictor of poor glycemic control. It emerged as the marker with better diagnostic accuracy showing high specificity and sensitivity in detecting poor glycemic control as compared to TAFI (Table 2). Also, in ROC analysis, the plasma level of PAI‐1 had a significantly high AUC, indicating its usefulness in predicting poor glycemic control. Since glycemic control and its predictors have become the topmost concern in the management of T2DM patients as morbidity and mortality in T2DM are believed to be due in large part to the direct consequences of chronic hyperglycemia, our results will provide a stimulus for further exploration of the potential discriminant predictive potential of PAI‐1 in this field.

## 5 CONCLUSION

T2DM is a hypercoagulable disorder with poor quality of glycemic control increasing the risk of thrombotic disorders due to hypofibrinolysis. This study, therefore, recommends the inclusion of a coagulation profile in the routine clinical assessments of patients with T2DM and the determination of genotypes of inhibitors of fibrinolysis in future studies.

## AUTHOR CONTRIBUTIONS

**Peter K. Selleh**: Conceptualization; data curation; investigation; methodology; resources; writing—review and editing. **Enoch O. Anto**: Conceptualization; data curation; investigation; methodology; supervision; writing—review and editing. **Wina I. O. Boadu**: Investigation; methodology; writing—original draft; writing—review and editing. **Benedict Sackey**: Conceptualization; investigation; methodology; supervision; writing—review and editing. **Lilian A. Boateng**: Conceptualization; investigation; methodology; supervision; writing—review and editing. **Charles Nkansah**: Data curation; methodology; software; validation; visualization; writing—review and editing. **Frederick Nsafoah**: Methodology; resources; validation; writing—review and editing. **Abdul R. Saasi**: Software; validation; visualization; writing—review and editing. **Selina Mintaah**: Data curation; investigation; writing—review and editing. **Yaw Amo Wiafe**: Investigation; methodology; resources; writing—review and editing. **Charles Derigubah**: Formal analysis; methodology; software; visualization; writing—review and editing. **Emmanuel E. Korsah**: Formal analysis; investigation; writing—original draft; writing—review and editing. **Joseph Frimpong**: Formal analysis; methodology; writing—original draft; writing—review and editing. **Ezekiel Ansah**: Data curation; formal analysis; software; writing—review and editing. **Valentine C. K. T. Tamakloe**: Software; visualization; writing—review and editing. **Patrick Adu**: Methodology; writing—review and editing. **Joseph Boachie**: Methodology; writing—review and editing. **Otchere Addai‐Mensah**: Conceptualization; project administration; supervision; writing—review and editing.

## CONFLICT OF INTEREST STATEMENT

The authors declare no conflict of interest.

## TRANSPARENCY STATEMENT

The lead author Otchere Addai‐Mensah affirms that this manuscript is an honest, accurate, and transparent account of the study being reported; that no important aspects of the study have been omitted; and that any discrepancies from the study as planned (and, if relevant, registered) have been explained.

## Data Availability

All data generated and analyzed during this study are included in the manuscript and can be requested from the corresponding author.
